# Predicting Pneumonia and Influenza Mortality from Morbidity Data

**DOI:** 10.1371/journal.pone.0000464

**Published:** 2007-05-23

**Authors:** Lise Denoeud, Clément Turbelin, Séverine Ansart, Alain-Jacques Valleron, Antoine Flahault, Fabrice Carrat

**Affiliations:** 1 Université Pierre et Marie Curie-Paris 6, UMR-S 707, Paris, France; 2 INSERM, U707, Paris, France; 3 Assistance Publique Hôpitaux de Paris, Hôpital Saint-Antoine, Paris, France; University of Cambridge, United Kingdom

## Abstract

**Background:**

Few European countries conduct reactive surveillance of influenza mortality, whereas most monitor morbidity.

**Methodology/Principal Findings:**

We developed a simple model based on Poisson seasonal regression to predict excess cases of pneumonia and influenza mortality during influenza epidemics, based on influenza morbidity data and the dominant types/subtypes of circulating viruses. Epidemics were classified in three levels of mortality burden (“high”, “moderate” and “low”). The model was fitted on 14 influenza seasons and was validated on six subsequent influenza seasons. Five out of the six seasons in the validation set were correctly classified. The average absolute difference between observed and predicted mortality was 2.8 per 100,000 (18% of the average excess mortality) and Spearman's rank correlation coefficient was 0.89 (P = 0.05).

**Conclusions/Significance:**

The method described here can be used to estimate the influenza mortality burden in countries where specific pneumonia and influenza mortality surveillance data are not available.

## Introduction

Influenza epidemics occur each winter, causing substantial morbidity and mortality worldwide [1]. The clinical severity of influenza increases with age and in individuals with underlying health disorders [2]. During seasonal epidemics more than 90% of all influenza-related deaths involve persons aged 65 years and more [3].

The mortality impact is highly variable from year to year [4]. The highest mortality rates typically occur in seasons when influenza A (H3N2) viruses predominate, rather than influenza A (H1N1) and B viruses [5].

Few European countries conduct reactive surveillance of influenza mortality, but most monitor influenza morbidity and virological characteristics [6]. Estimates of the influenza mortality burden generally become available 2–3 years after the corresponding epidemic and are usually based on post-hoc analysis of national pneumonia and influenza (P&I) mortality statistics. For public health purposes, knowledge on influenza mortality can have important practical implications. Firstly, people and particularly the representatives of health authorities “want to know”. Secondly, real-time predictions of mortality could theoretically help planning hospitals needs or deciding on antiviral or vaccine interventions.

Here we describe a simple model designed to predict the weekly excess of P&I mortality in persons aged 65 years or more, based on influenza morbidity data and the types/subtypes of circulating viruses.

## Methods

The weekly numbers of P&I deaths in France were obtained from death certificates collected by the *Centre d'épidémiologie sur les causes médicales de décès* between October 1984 and December 2004. We identified P&I deaths in persons aged 65 years and older by using codes 480–487 of the International Classification of Diseases (ICD)-9^th^ revision up to 31 December 1999, and codes J10–J11, J12–J18 of the ICD-10^th^ revision after 1 January 2000.

Morbidity data were obtained after 1984 from the *Sentinelles* system, [www.sentiweb.org] [7], a French nationwide network of general practitioners who report, in real time, the number of consultations for influenza-like illness (ILI). ILI is defined as sudden-onset fever above 39°C with respiratory symptoms and stiffness or myalgias. The age, sex, vaccination status and location of cases is also reported. By using population data from regular censuses [http://www.insee.fr/fr/ffc/ipweb/ip1089/ip1089.xls], we calculated weekly ILI incidence rates. The weekly proportion of patients over 65 years was used as a proxy for the ILI age distribution.

For each influenza season, we extracted from available data ([8] and the European Influenza Surveillance Scheme [www.eiss.org]) the dominant types and subtypes of circulating influenza viruses in France (one or two types per epidemic).

We first applied a Poisson seasonal regression model to morbidity data in order to estimate the baseline seasonal ILI incidence [9], [10]: the model was estimated based on non-epidemic data, defined as a weekly morbidity rate below 279 cases per 100,000 inhabitants. Seasonality was modeled by introducing periodic functions of time as covariates. The model also included a linear function of time for capturing the secular time trend in the data that may be due to demographic changes (e.g. population aging). Predicted baseline seasonal ILI incidence rates were used to define epidemic periods, i.e. when the observed ILI rate exceeded the 95 percent confidence limit of the predicted baseline ILI rate for two consecutive weeks. The difference between observed and predicted baseline ILI rates during epidemic periods was used to define excess ILI morbidity.

We then applied a similar strategy to P&I mortality data: We fitted a Poisson seasonal regression model to weekly mortality data in persons aged 65 years or more, excluding deaths during ILI epidemic periods. We extended the ILI epidemic periods by two weeks in order to deal with the potential delay between symptom onset and death attributable to influenza. A dummy variable was introduced to take into account the change in the version of the ICD classification [11], so that we could estimate in the model the conversion factor resulting from this modification. Excess P&I mortality was calculated as the difference between observed P&I mortality and predicted baseline seasonal mortality, during epidemic weeks. Epidemics were further classified in three levels of mortality burden (“high”, “moderate” and “low”) according to the tertiles of the observed excess mortality distribution.

Finally, we fitted a Poisson regression model predicting weekly excess P&I mortality in persons aged 65 years or more from the weekly excess ILI morbidity, the weekly proportion of cases aged 65 years or more out of all ILI , and the dominant influenza virus types or subtypes (A/H3N2, A/H1N1, B). The model was estimated over 14 winter periods between October 1984 and October 1998, and validated over 6 winter periods between October 1998 and December 2004. The conversion factor estimated from the mortality seasonal model was introduced to correct estimations after year 2000. We tested morbidity and mortality data simultaneously, lagged by one week, and lagged by two weeks, and we selected the best model. Deviance statistics were used for model selection, and parameters were tested using the Wald chi-square method. Average absolute differences between observed and predicted values and Spearman's rank correlations were used to assess predictability. All analyses were done with SAS software, version 9.1 (SAS institute, Cary, NC).

## Results

**Figure 1:**
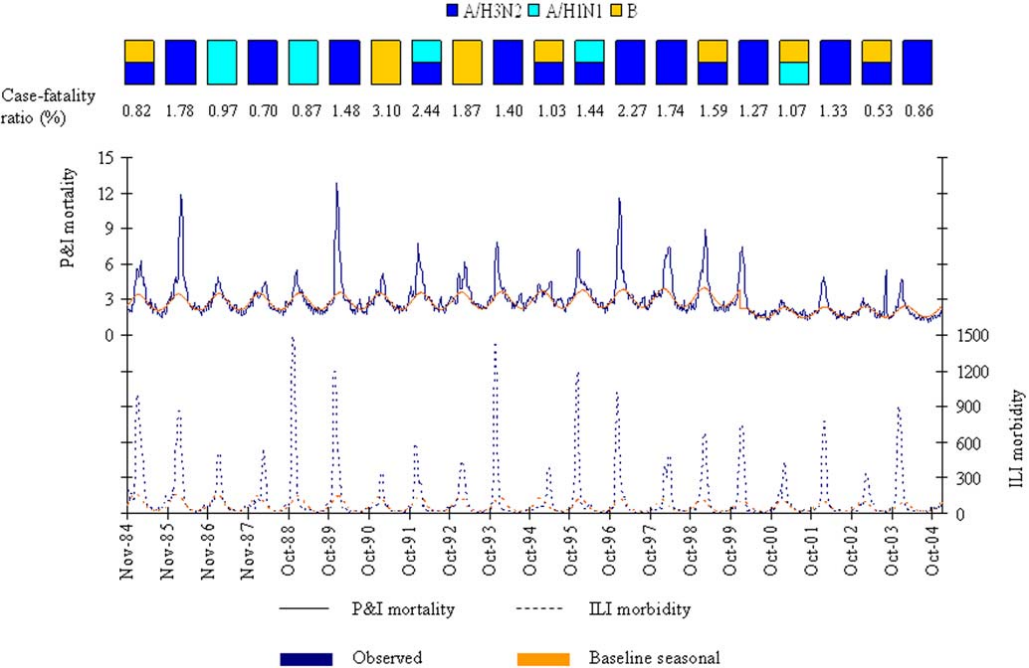
Observed and baseline seasonal rates of P&I mortality^a^ and ILI morbidity^b^. The dominant types and subtypes of circulating influenza viruses in France, 1984–2004, are shown in the rectangles. The case-fatality ratio of influenza is defined as the ratio of cumulated excess mortality to cumulated excess morbidity in people over 65 years of age. P&I: pneumonia and influenza. ILI: influenza-like illness. ^a^ rates per week and per 100,000 inhabitants over 65 years of age. ^b^ rates per week and per 100,000 inhabitants.

The P&I mortality, ILI morbidity weekly data and the dominant types/subtypes of circulating influenza viruses are given Figure 1. The highest mortality rate was observed in winter 1989/1990, when A/H3N2 viruses were circulating. The highest ILI morbidity rate was observed during winter 1988/1989, when A/H1N1 viruses were circulating.

Between 1984 and 2004 the model of baseline seasonal ILI morbidity identified 20 epidemic periods, observed from the second week of November to the first week of May, and lasting from 6 to 15 weeks. Excess ILI morbidity ranged from 0 to 1387 cases per 100,000 inhabitants per week, with a median of 222 per 100,000 inhabitants per week.

Excess P&I mortality ranged from 0 to 9.4 per 100,000 inhabitants over 65 years of age and per week (during winter 1989/1990), with a median of 1.4 deaths per 100,000 inhabitants per week. The conversion factor considering the change in ICD classification was estimated to 0.57 (95% CI 0.56–0.58) in the seasonal model. The case-fatality ratio of influenza, defined here as the ratio of cumulated excess mortality to cumulated excess morbidity in people over 65 years of age, ranged from 0.53% for the 2002/2003 epidemic to 3.10% for the 1990/1991 epidemic. The average case-fatality ratio was 1.43% for A/H3N2 viruses, 1.36% for A/H1N1 and 1.38% for B viruses (P = 0.12, Kruskall-Wallis test).

**Figure 2:**
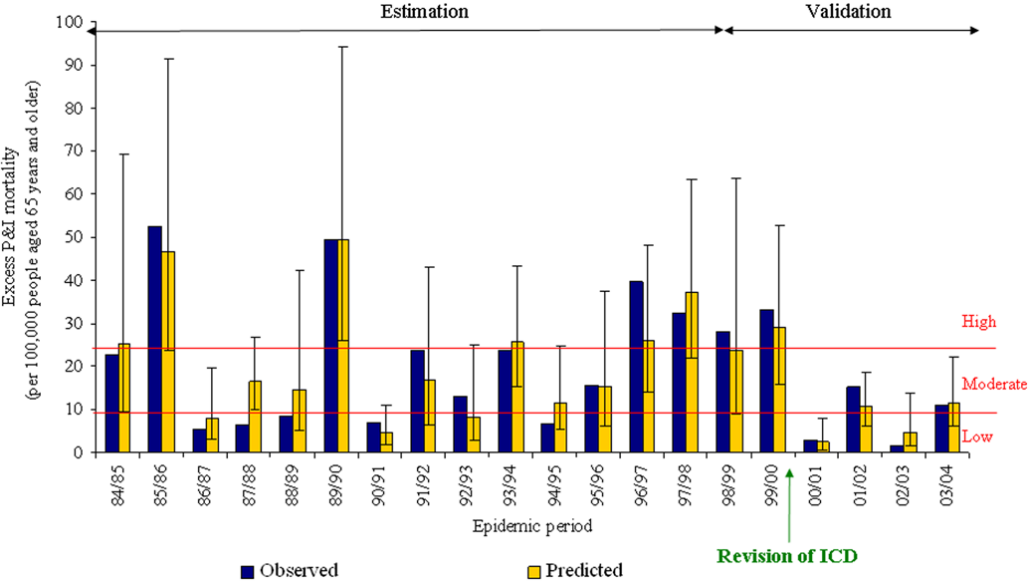
Observed and predicted excess P&I mortality cumulated by epidemic periods in persons over 65 years. Predicted values are presented with their 95% confidence intervals. The red lines represent the tertiles of the observed P&I mortality. P&I: pneumonia and influenza. ICD: International Classification of Diseases.

One hundred and forty-five weeks were used to estimate the P&I mortality Poisson predictive model, and 70 weeks were used for validation (Figure S1). The best selected model used excess ILI morbidity (P<0.001), and the proportion of cases aged 65 years or more out of all ILI (P<0.001) both lagged by one week. A dominant type B influenza virus was associated with less excess mortality (P = 0.023). No association was found between the proportion of cases aged 65 years or more out of all ILI and the dominant types or subtypes of circulating influenza viruses (P = 0.13, Kruskall-Wallis test). Cumulated excess P&I mortality was classified in three tertiles, using 9.4 and 23.9 deaths per 100,000 inhabitants over 65 years as cut-off values. The model adequately fitted P&I mortality (Figure 2). The average absolute differences between observed and predicted values were 4.8 deaths per 100,000 inhabitants (minimum 0.2, maximum 13.8, 22% of the average observed excess) in the estimation set, and 2.8 (minimum 0.4 maximum 4.6, 18 % of the average observed excess) in the validation set. The rank correlation between observed and predicted values was 0.86 (P = 0.001) in the estimation set and 0.89 (P = 0.05) in the validation set. Five out of six epidemics of the validation set (one high, two moderate, and two low) were correctly predicted. A discrepancy between the observed (28.1, “high”) and the predicted (23.8, “moderate”) burden was obtained for the 1998/1999 epidemic, but the absolute difference was small.

## Discussion

Our simple seasonal regression model based on morbidity and virological surveillance data provides accurate estimates of pneumonia- and influenza-related mortality. Although a previous study linked influenza morbidity to influenza mortality [12], ours is the first to link excess influenza morbidity, i.e. morbidity above the baseline observed during influenza epidemic periods, with excess P&I mortality. Pneumonia is a frequent complication of influenza and most influenza deaths result from secondary bacterial pneumonia. Excess P&I mortality is a widely used and sensitive indicator of influenza-associated death. [9]. This is why we grouped pneumonia and influenza deaths together, instead of treating them separately.

Our model does not use quantitative virological surveillance data, which are available in several European countries. On the other hand, laboratory testing for influenza infection is rare in case of respiratory death, and many other respiratory viruses (e.g. respiratory syncitial virus, human metapneumoviruses) may contribute to P&I deaths [13]. In order to preserve consistency between ILI and P&I, and also for reasons of simplicity, timeliness and ease of access, qualitative information on the dominant influenza virus type was used instead of quantitative virological data. Diagnostic uncertainty in ILI as well as in P&I mortality may increase the discrepancies between observed and predicted P&I mortality.

Influenza is known to increase all-cause mortality [14]–[16]. We did not try to predict all-cause influenza-associated mortality, but a similar methodological approach could be used for this purpose.

Finally, we did not introduce influenza vaccine coverage (or effectiveness) in people over 65 years of age. Although influenza vaccine may prevent ILI or P&I deaths we postulated that vaccination would not prevent influenza deaths without preventing influenza. Thus predictions of P&I deaths from ILI would not be affected by introducing influenza vaccination in the model.

In countries where influenza morbidity and virological data are the focus of reactive surveillance, our method could be used to predict excess P&I mortality almost in real time. It could guide prevention strategies, when the P&I mortality is coming to an alert level.

## Supporting Information

Figure S1 Weekly observed and predicted excess P&I mortality in persons over 65 years. P&I: pneumonia and influenza
(0.06 MB TIF)
