# Supplementary figures and images for: Predicting Pneumonia and Influenza Mortality from Morbidity Data
Source: PLoS One. 2007 May 23;2(5):e464. doi: 10.1371/journal.pone.0000464 (PMC1866180; doi:10.1371/journal.pone.0000464)

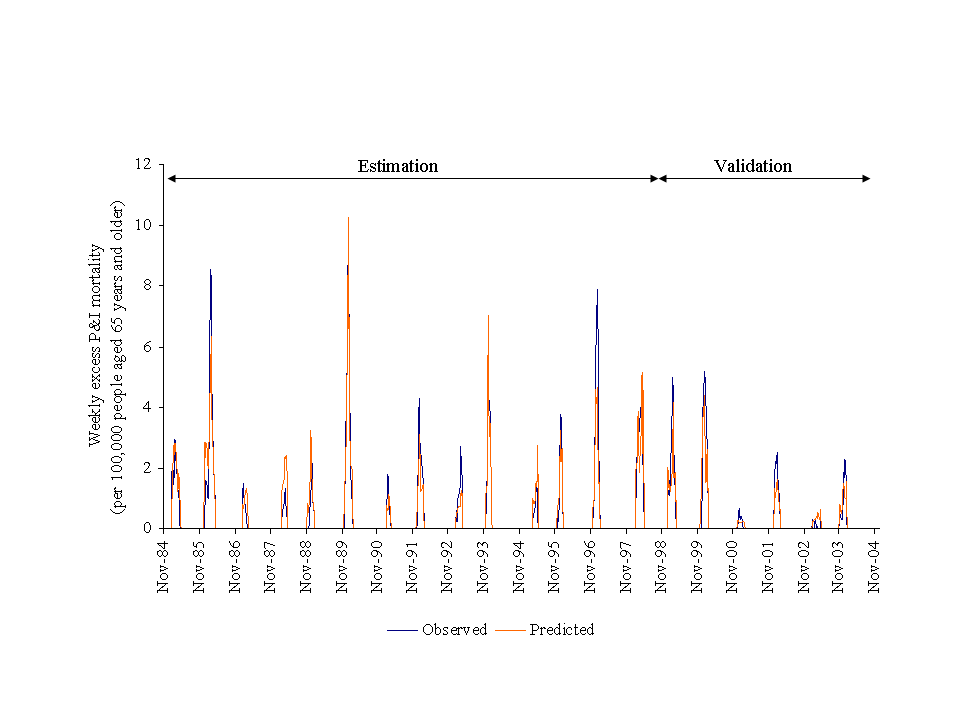

Supplement: Figure S1 — Weekly observed and predicted excess P&I mortality in persons over 65 years. P&I: pneumonia and influenza (0.06 MB TIF) [file pone.0000464.s001.tif]
